# Cold-Induced Changes in Gene Expression in Brown Adipose Tissue, White Adipose Tissue and Liver

**DOI:** 10.1371/journal.pone.0068933

**Published:** 2013-07-22

**Authors:** Andrew M. Shore, Angeliki Karamitri, Paul Kemp, John R. Speakman, Neil S. Graham, Michael A. Lomax

**Affiliations:** 1 School of Biosciences, Cardiff University, Cardiff, United Kingdom; 2 Inserm, Institut Cochin, Paris, France; 3 National Heart and Lung Institute, Imperial College London, London, United Kingdom; 4 School of Biological Sciences, University of Aberdeen, Aberdeen, United Kingdom; 5 School of Biosciences, University of Nottingham, Loughborough, United Kingdom; Boston University Medical Center, United States of America

## Abstract

Cold exposure imposes a metabolic challenge to mammals that is met by a coordinated response in different tissues to prevent hypothermia. This study reports a transcriptomic analysis in brown adipose tissue (BAT), white adipose (WAT) and liver of mice in response to 24 h cold exposure at 8°C. Expression of 1895 genes were significantly (P<0.05) up- or down-regulated more than two fold by cold exposure in all tissues but only 5 of these genes were shared by all three tissues, and only 19, 14 and 134 genes were common between WAT and BAT, WAT and liver, and BAT and liver, respectively. We confirmed using qRT-PCR, the increased expression of a number of characteristic BAT genes during cold exposure. In both BAT and the liver, the most common direction of change in gene expression was suppression (496 genes in BAT and 590 genes in liver). Gene ontology analysis revealed for the first time significant (P<0.05) down regulation in response to cold, of genes involved in oxidoreductase activity, lipid metabolic processes and protease inhibitor activity, in both BAT and liver, but not WAT. The results reveal an unexpected importance of down regulation of cytochrome P450 gene expression and apolipoprotein, in both BAT and liver, but not WAT, in response to cold exposure. Pathway analysis suggests a model in which down regulation of the nuclear transcription factors HNF4α and PPARα in both BAT and liver may orchestrate the down regulation of genes involved in lipoprotein and steroid metabolism as well as Phase I enzymes belonging to the cytochrome P450 group in response to cold stress in mice. We propose that the response to cold stress involves decreased gene expression in a range of cellular processes in order to maximise pathways involved in heat production.

## Introduction

Cold exposure imposes a metabolic challenge to mammals that must be met by a coordinated response in different tissues to ensure homeothermy. Most mammals possess a specialized thermogenic tissue, brown adipose tissue (BAT) which is activated by the sympathetic nervous system during cold exposure and produces heat as a result of the BAT-specific expression of uncoupling protein 1 (UCP-1) [1]. Both BAT and white adipose tissue (WAT) possess a store of triacylglycerol (TAG) containing lipid droplets which are mobilised to provide fatty acids as a fuel for thermogenesis induced by the uncoupling of oxidative phosphorylation in BAT by UCP-1 [2]. Additionally glucose acts as a thermogenic fuel in BAT and cold stress results in increased hepatic gluconeogenesis as well as increased insulin sensitivity [3]. Therefore, the provision of substrate fuels for the thermogenic metabolic response to cold exposure requires a coordinated response in BAT, WAT and liver.

Previous studies have established preferential expression of genes involved in fatty acid metabolism in BAT compared to WAT [4]–[7]. The expression of genes involved thermogenic function of BAT are increased by 48 h of cold exposure [8]–[10]. By contrast cold elicited fewer and more moderate changes in gene expression in WAT [11], the only response being increased UCP1 expression which is now recognized as being the result of changes in brown adipocytes in WAT (BRITE cells) [12]. Despite the importance of the liver to metabolic adaptation there have been no studies, to our knowledge, describing the effect of cold on gene expression in this tissue in mammals, although this approach has been taken in fish which have a very different thermal physiology [13], [14]. Here we report an analysis of transciptome profiles in the three tissues in response to 24 h cold exposure in mice.

## Materials and Methods

### Animal Experiments

Two groups of C57BL/6 mice were used each consisting of four female individuals. Both groups were housed individually in cages measuring 48×15×13 cm at 16 h light and 8 h dark cycle with access to bedding material. All groups had access ad libitum to a standard mouse chow diet. Mouse weight and feed consumption was measured at 24 h intervals. One group was kept at 22°C ±2°C for 72 h; these mice comprised the room temperature acclimatised group. A second group was kept at 22°C ±2°C for 48 h followed by 8°C ±2°C for 24 h and comprised the cold acclimatised group. The experimental protocol was approved by the Ethical Review Committee (ERC) of the College of Life Sciences and Medicine at the University of Aberdeen, and subsequently carried out under licence from the UK Home Office. Between 1200 and 1600 h, animals were euthanised by concussion and cervical dislocation following Home Office guidelines. Samples of liver, white and brown adipose tissue were taken from each animal and immediately frozen in liquid nitrogen followed by storage at −80°C prior to analysis.

### Quantitative Real Time PCR

Total RNA was extracted from each tissue from all four animals in each of the two temperature groups (24 samples in total) using TRI reagent (Sigma). Prior to RT-PCR, samples were treated with RNase-free DNase to remove contaminating genomic or plasmid DNA. cDNA was generated using the cDNA synthesis kit from Qiagen. Quantitative Real Time PCR (qRT-PCR) was performed using Sybr green (Qiagen) according to the manufacturer’s instructions in Rotor Gene 3000 (Corbert Research). The sequences of the primers used for Real Time PCR were: PGC-1α sense GCGCCGTGTGATTTACGTT and antisense AAAACTTCAAAGCGGTCTCTCAA, UCP1 sense CCTGCCTCTCTCGGAAACAA antisense TGTAGGCTGCCCAATGAACA, C/EBPβ sense GCAAGAGCCGCGACAAG antisense GGCTCGGGCAGCTGCTT, PPARα sense TGCCAGTACTGCCGTTTTCA antisense GCGAATTGCATTGTGTGACAT, HNF4α sense GTCGAGTGGGCCAAGTACATC antisense CGCCACCTGGTCATCCA, 18S rRNA sense GTAACCCGTTGAACCCCATT and antisense CCATCCAATCGGTAGTAGCG and 36B4 sense TCCAGGCTTTGGGCATCA and antisense TTATCAGCTGCACATCACTCAGAAT. Expression levels for all genes were normalized to the internal control 18s rRNA and 36B4.

### Illumina Microarray

For each of the three tissues, RNA samples from 3 animals at room temperature and 3 animals in the cold environment (total 18 samples handled separately), were labeled and amplified using the Illumina RNA Amplification Kit, and then hybridized to the MouseWG-6_V1 array using standard protocols. The data discussed in this publication have been deposited in NCBI's Gene Expression Omnibus [15] and are accessible through GEO Series accession number GSE44138 (http://www.ncbi.nlm.nih.gov/geo/query/acc.cgi?acc=GSE44138)”.

All data were analysed in Genespring GX (version 10, Agilent Tehnologies). Normalized signal values for individual probes were standardized to the median signal value for that probe across all samples. Probes were further filtered based on a flag call of present or marginal in 2 of 17 samples (lower cut-off for present = 0.8, upper cut-off for absent call = 0.6). Analysis on one liver sample failed resulting in only two animals in the control and cold exposed groups being analysed for this tissue.

Identification of differentially expressed genes and tissue specific gene expression was performed by hierarchical clustering of the samples using Genespring GX, employing a Euclidean distance matrix and Centroid linkage rule. K-means clustering was performed employing a Euclidean distance matrix using 6 clusters and 200 iterations. Principal component analysis was performed using Genespring GX using default parameters. Differentially expressed genes between treated and control samples from each tissue were identified using a T-test, p≤0.05 and a fold change cut-off ≥2.0. Genes that were differentially expressed in a single tissue were identified using a Venn diagram (using Genespring GX) of the differentially expressed genes in each tissue. GO analysis was performed using the GO analysis function in Genespring GX and over-representation of GO terms was identified using a hypergeometric test with a Benjamini-Yekutieli corrected p-value ≤0.05.

## Results

### Gene Expression Responses to Cold

**Figure 1:**
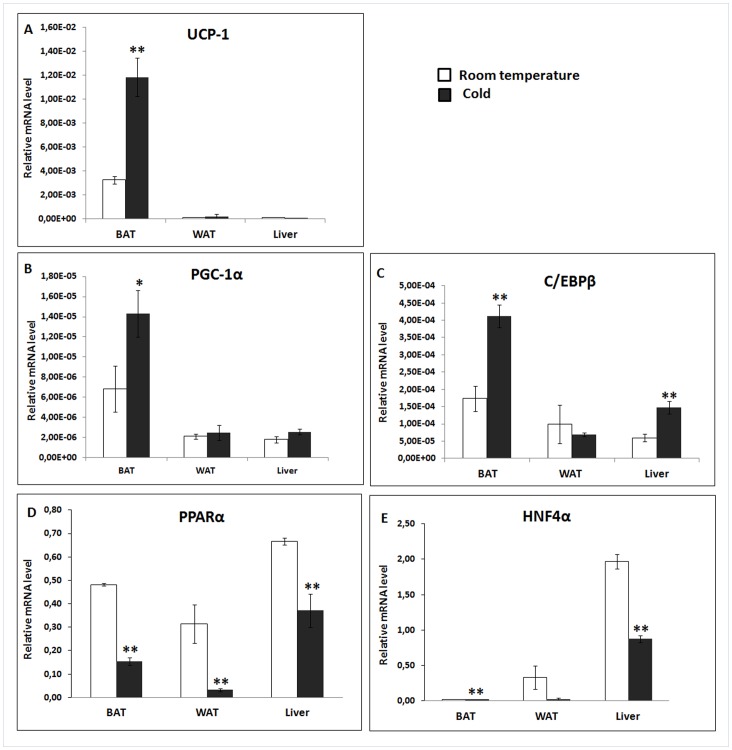
Expression of (A) UCP1, (B) PGC-1α, (C ) C/EBPβ, (D) PPARα and (E) HNF4α in brown adipose tissue (BAT), white adipose tissue (WAT) and liver of acutely cold stressed mice. Animals were maintained at 22°C ±2°C (room temperature) or at 8°C ±2°C for 24 h (cold). Tissue samples were collected, mRNA prepared and analyzed by qRT-PCR. Values are normalised against either 18S rRNA or 36B4 expression and represent means +/− SEM of four animals in each group.

To confirm that there was a significant response in the mice to a 24 h of cold exposure, we analysed the expression of two genes (UCP-1 and PGC1α) by qRT-PCR in RNA isolated from BAT. This analysis showed a significant increase in the expression of both genes (P<0.05, UCP1; 4 fold; PGC1α, 2 fold; Figure 1A, 1B) consistent with the known response of BAT to cold exposure [16]–[18].

**Figure 2:**
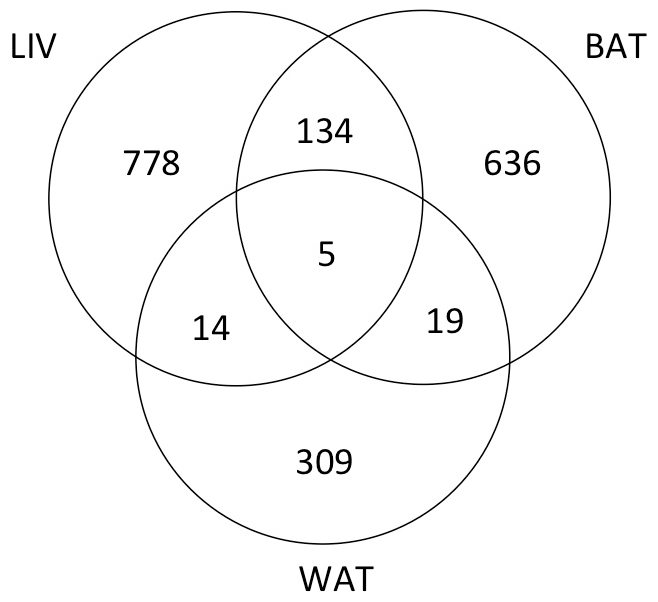
Venn diagram showing the overlap in genes differentially expressed between the cold and room temperature treatments for brown adipose tissue (BAT), white adipose tissue (WAT) and liver (LIV). Animals were maintained at 22°C ±2°C (room temperature; 3 animals) or at 8°C ±2°C for 24 h (cold; 3 animals). Tissue samples were collected, mRNA prepared and analyzed by Illumina microarray as described in the text. Differentially expressed genes (≥2 fold, P<0.05) between cold and room temperature treated values were identified using Genespring GX.

**Figure 3:**
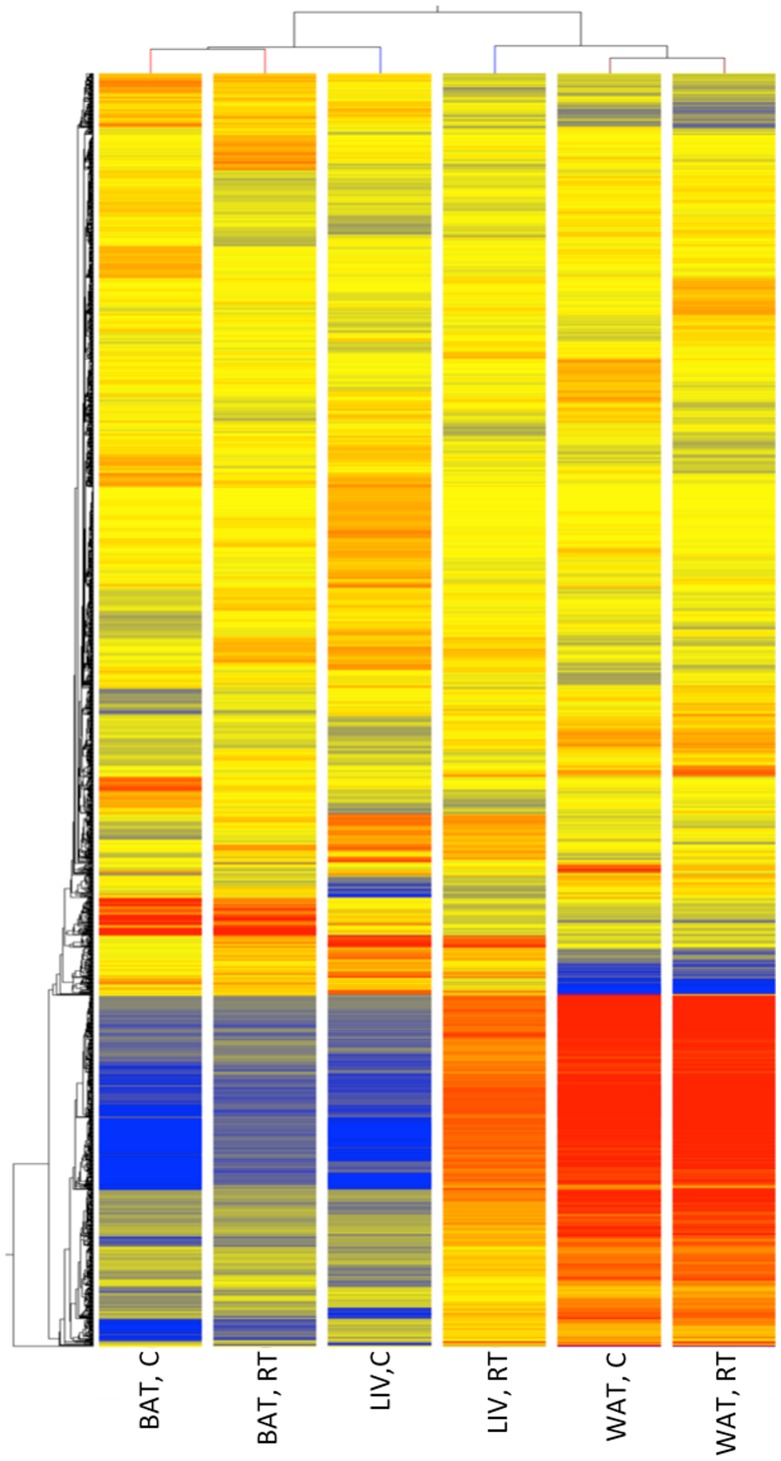
Hierarchical clustering of differentially expressed genes between the cold and room temperature treatments. The columns represent samples of white adipose tissue (WAT), brown adipose tissue (BAT) and liver (LIV) at room temperature (RT) or cold treated (C). The rows represent the 1895 differentially expressed probe-sets between treatments in all tissues, which are coloured based on their normalised signal values (red - high, blue = low).

**Figure 4:**
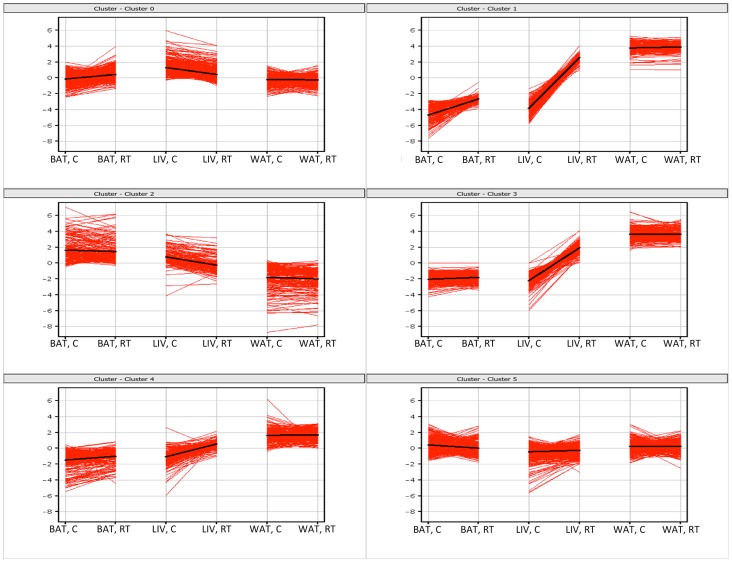
K-means clustering of the differentially expressed genes between the cold and room temperature treatments. The x-axis represent samples of white adipose tissue (WAT), brown adipose tissue (BAT) and liver (LIV) at room temperature (RT) or cold treated (C) and y-axis the normalised signal level. The lines represent the 1895 differentially expressed probe-sets between treatments in all tissues. The black line represents the mean profile of each cluster.

Having established a cold response in the mice we analysed transcript profiles in all three tissues (BAT, WAT and liver) using microarrays. This analysis (Figure 2 and Tables S2, S3 and S4) showed that the expression of 1895 genes were significantly (P<0.05) up- or down-regulated more than two fold by cold exposure in at least one tissue. Of these genes, all three tissues shared only 5 gene transcript responses to cold. Comparing the tissues in a pair wise manner, 19, 14 and 134 gene expression changes were common between WAT and BAT, WAT and liver, and BAT and liver, respectively (Figure 2). The number of genes in which expression was significantly altered in only one tissue was 636 in BAT, 309 in WAT, and 778 in liver. Cluster analysis of the 1895 differentially expressed genes (Figures 3 and 4) clearly showed that gene expression responses to cold stress were tissue specific, as gene clusters are not identified consisting of genes that responded to cold in all tissues.

Although the precise changes in gene expression were tissue specific, gene ontogeny (GO) analysis (Table 1) revealed a remarkable similarity between the gene groups responding to the cold environment in BAT and in liver. In both tissues, genes associated with oxidoreductase activity, Fe^3+^ binding, endoplasmic reticulum function lipid metabolic processes and protease inhibitor activity were highly represented. GO analysis failed to reveal any significant functional gene groups in the genes changing in response to cold only in WAT.

**Table 1:** Over-represented GO terms in differentially expressed genes of BAT and Liver.

Brown OnlyGO ACCESSION	GO Term	Genes in listin category	% of genes in listin category	p-value
GO:0016491	oxidoreductase activity	27	42.2	1.30E-07
GO:0005506	iron ion binding	17	26.6	2.90E-06
GO:0042598	endoplasmic reticulum	12	18.8	4.54E-06
GO:0030414	protease inhibitor activity	7	10.9	1.67E-05
GO:0044255	cellular lipid metabolic process	11	17.2	7.09E-05
Liver Only				
GO:0016491	oxidoreductase activity	40	25	7.84E-11
GO:0005506	iron ion binding	20	12.5	1.46E-06
GO:0005783	endoplasmic reticulum	39	24.4	5.27E-09
GO:0004866	endopeptidase inhibitor activity	6	3.75	4.91E-04

In both BAT and the liver the largest and most common direction of change in gene expression was suppression with 496 genes reduced in BAT and 590 genes reduced in liver. 28 and 37 genes associated with oxidoreductase activity (Table S1) were suppressed in BAT and liver, respectively, 16 and 22 genes associated with lipid metabolic processes (Table 2) were suppressed in BAT and liver, respectively, and 11 and 10 genes associated with protease inhibitor activity (Table 3) were suppressed in BAT and liver, respectively. If these observations were converted into changes in protein it sugsgests that there was a tendency to limit energy expenditure on a range of metabolic processes to maximise energy expenditure on heat production, although this suggestion requires analysis that is beyond the scope of our data set.

**Table 2:** GO Lipid Metabolic Process list of genes up regulated and down regulated genes all tissues.

Lipid Metabolic Process			Fold change		Fold change		Fold change	
Symbol	Definition_1	Accession	Brown	Regulation	Liver	Regulation	White	Regulation
			Fold change		Fold change		Fold change	
Elovl3	elongation of very long chain fatty acids	NM_007703.1	7.94	up	1	down	1.17	Down
Stard5	StAR-related lipid transfer (START) domain containing 5	NM_023377.4	2.31	up	3.65	down	1.15	Up
Stard5	StAR-related lipid transfer (START) domain containing 5	NM_023377.4	2.10	up	2.77	down	1.04	Down
Acot8	acyl-CoA thioesterase 8	NM_133240.1	2.09	up	1.28	down	1.02	Down
Il4	interleukin 4	NM_021283.1	2.07	up	1.11	down	1.15	Up
Mvd	mevalonate (diphospho) decarboxylase	NM_138656.1	2.03	up	1.14	down	2.18	Up
Apoa2	apolipoprotein A-II	NM_013474.1	26.95	down	44.48	down	1.02	Up
Apoa2	apolipoprotein A-II	NM_013474.1	17.51	down	189.50	down	2.71	Down
Apof	apolipoprotein F	NM_133997.1	12.32	down	77.66	down	1.21	Up
Apoa1	apolipoprotein A-I	NM_009692.1	8.28	down	141.26	down	1.26	Down
Hmgcs2	3-hydroxy-3-methylglutaryl-Coenzyme A synthase 2	NM_008256.2	7.89	down	34.58	down	1.07	Up
Apoc3	apolipoprotein C-III	NM_023114.2	6.16	down	194.50	down	1.13	Down
Nr1h4	nuclear receptor subfamily 1, group H, member 4	NM_009108.1	5.53	down	42.22	down	1.13	Down
Azgp1	α-2-glycoprotein 1, zinc	NM_013478.1	4.52	down	139.00	down	1.27	Down
Cyp7a1	cytochrome P450, family 7, subfamily a, polypeptide 1	NM_007824.2	3.35	down	45.55	down	2.66	Up
PPARα	peroxisome proliferator activated receptor α	NM_011144.2	3.01	down	3.52	down	1.18	Down
Sgms2	sphingomyelin synthase 2	NM_028943.1	2.77	down	1.28	down	1.38	Down
Cyb5r3	cytochrome b5 reductase 3	NM_029787.2	2.69	down	1.55	down	1.67	Down
Cryl1	crystallin, lambda 1	NM_030004.2	2.54	down	1.88	down	1.24	Down
Adh1	alcohol dehydrogenase 1	NM_007409.2	2.24	down	11.16	down	1.51	Down
Ptgis	prostaglandin I2 (prostacyclin) synthase	NM_008968.2	2.23	down	1.12	down	2.16	Up
Fads1	Fatty acid desaturase 1	NM_146094.1	2.00	down	3.08	down	1.12	Up

**Table 3:** GO Protease Inhibitor List of up regulated and down regulated genes all tissues.

Protease Inhibitor List			Fold change		Fold change		Fold change	
Symbol	Definition_1	Accession	Brown	Regulation	Liver	Regulation	White	Regulation
Mug4	murinoglobulin 4	NM_008645.2	22.68	down	216.19	down	1.28	Down
Ahsg	α-2-HS-glycoprotein	NM_013465.1	11.96	down	304.21	down	1.05	Down
Itih4	inter α-trypsin inhibitor, heavy chain 4 (	NM_018746.1	10.82	down	168.43	down	1.04	Up
Serpinf2	serine (or cysteine) peptidase inhibitor, clade F, member 2	NM_008878.1	10.51	down	202.53	down	1.02	Down
Itih2	inter-α trypsin inhibitor, heavy chain 2 (Itih2).	NM_010582.1	8.49	down	109.37	down	1.16	Down
Serpina3m	serine (or cysteine) peptidase inhibitor, clade A, member 3M	NM_009253.1	6.30	down	26.97	down	1.20	Up
Serpina10	serine (or cysteine) peptidase inhibitor, clade A	NM_144834.2	4.72	down	44.61	down	1.02	Down
Fetub	fetuin beta	NM_021564.1	4.71	down	102.29	down	1.36	Down
Serpina3n	serine (or cysteine) peptidase inhibitor, clade A, member 3N	NM_009252.1	2.39	down	1.06	down	1.13	Down
Mug1	murinoglobulin 1	NM_008645.2	2.10	Down	71.52	down	1.00	Up
Serpine2	serine (or cysteine) peptidase inhibitor, clade E, member 2	NM_009255.2	2.02	Down	1.22	up	1.39	Up

### Gene Expression Responses to Cold in Brown Adipose Tissue

Analysis of the changes in gene expression in BAT confirmed and extended previous microarray-based and qRT-PCR-based studies which have catalogued the gene expression changes in BAT during cold adaptation (Table S2). As previously described, exposure to the cold environment, increased expression of type II iodothyronine deiodinase (DIO; 7.6 fold), Elovl3 (8.0 fold) and glycerol kinase (GK;6.6 fold) [19]-[21] in BAT. Other changes in gene expression of particular note in BAT were a significant increase in the expression of the transcription factor C/EBPβ (1.4 fold P<0.037; Figure 1C) and a significant (3.0 fold, p<0.01) reduction in the expression of PPARα (Figure 1D). Although we observed a significant change in UCP1 and PGC1α by qRT-PCR (Figure 1A, 1B) no changes were seen in the expression of UCP1 and only a modest not statistically significant change (1.8 fold) in PGC1α was detectable in the array data. Previous studies have also failed to report an increase in the transcript for UCP1 in BAT in response to cold due to the high signal level in this tissue [22]. There was an increase in the expression of a number of genes associated with mitochondrial function including 4 encoding components of mitochondrial ribosomal proteins (MrpL3, MrpL15, MrpL20 and MrpL51) and a protein involved in mitochondrial ribosome recycling (Mrrf). We did not observe any change in the expression of genes directly involved in mitochondriogenesis (e.g. cytochrome c; cox8b) although there was an increase in mitochondrial DNA (mtDNA)-encoded subunits of complex I (mtDNA ND3;3.4 fold). Nuclear hormone receptor genes were significantly altered both upwards (Nr1i3, 1.6 fold; Rxrb 1.6 fold, ESrrb 3.1 fold; Nr4a2 2.7 fold) and downwards (PPARα 3 fold; Nr1h4 5.5 fold). Surprisingly there were no changes in genes involved in BAT differentiation (e.g.CideA, PRDM16).

### Gene Expression Responses to Cold in White Adipose Tissue

Cold exposure has been reported to increase glucose utilisation and insulin sensitivity in WAT [23]. In the present study cold induced a significant increase in insulin receptor expression (2.2 fold) and a significant down regulation of TRIB3 (4.0 fold), a negative regulator of the Akt, which has been associated with improved adipose insulin sensitivity (Table S3) [24], [25]. Cold would be expected to increase lipolysis in WAT but there were no significant changes in genes regulating lipid synthesis of breakdown. There was significant up regulation of hydroxy-delta-5-steroid dehydrogenase (2.2 fold), a gene responsible for steroid biosynthesis and thyroid deiodinase I (Dio 1; 4.7), a gene linked to increased thyroid action on cellular respiration.

### Gene Expression Responses to Cold in Liver

A feature of the hepatic gene expression response to cold was the significant suppression of a large number of genes involved in maintaining cell metabolism (Table S4). The cytochrome P450 oxidoreductase group of enzymes was particularly well represented in this group with 17 genes being decreased 6.5–281 fold. A number of genes coding for enzymes catalysing oxido reductase reaction were suppressed: hydroxy-delta-5-steroid dehydrogenase (96.4 fold); retinol dehydrogenase (140 fold); proline dehydrogenase (130 fold); butyrobetaine (gamma), 2-oxoglutarate dioxygenase (59 fold); sorbitol dehydrogenase (5.95 fold); aldo-keto reductase (3.9 fold); aldehyde dehydrogenase (3.2 fold); lactate dehydrogenase (2.8 fold) although NADH dehydrogenase (ubiquinone) (3.8 fold); and malate dehydrogenase 2 (3,3 fold) were up regulated. Similarly, oxidase enzymes were generally suppressed: urate oxidase (267 fold); gulonolactone (L-) oxidase (58.6 fold); flavin containing monooxygenase 5 (23.9 fold); pipecolic acid oxidase (39.1 fold) although monoamine oxidase (16.1 fold) and glutathione peroxidise 8 (2.1 fold) were upregulated.

Surprisingly, given the expected increase in oxidative metabolism in a cold environment, genes involved in lipid metabolic reactions were down regulated including apolipotrotein AI, AII, AIV, B, CII, CIII, CIV, M (77–319 fold); fatty acid desaturase 1 (3.08); StAR-related lipid transfer (3.65 fold); Analysis of the changes in gene expression in the liver identified significant reduction in the expression of a number of genes associated with cholesterol and bile transport and metabolism: ATP binding cassette transporters which regulate cholesterol efflux (27–29 fold), cholesterol 7-hydroxylase (45 fold) and 3-hydroxy-3-methylglutaryl-Coenzyme A synthase 2 (34.6 fold), which regulates bile acid synthesis from cholesterol, sodium,/bile acid cotransporter (171.5 fold) and the bile acid metabolising enzyme CY8B1 (88 fold), bile acid-Coenzyme A: amino acid N-acyltransferase (13.5 fold) and bile acid CoA ligase (125 fold).

The exposure to a cold environment also suppressed hepatic expression of a group of 10 genes with protease inhibitor function including murinoglobulin 1 & 4 (216.4 fold), α-2-HS-glycoprotein (304.2 fold), Serpin 2,3 and 10 (27.0–202.5 fold), inter-α trypsin inhibitor, heavy chain 2 & 4 (109.4–168.4 fold), fetuin beta (102.3 fold). Expression of urea cycle enzymes were decreased by cold stress; argininosuccinate lyase (12.1 fold); argininosuccinate synthetase 1(42 fold); carbamoyl-phosphate synthetase 1 (167 fold); mitochondrial carrier ornithine transporter (7.9 fold); the urea cycle regulator N-acetylglutamate synthase (23 fold). A number of genes in the glycolytic/gluconeogenic pathways were also suppressed: hexokinase (26.7 fold), glucokinase (43.0 fold), glucokinase regulatory protein (30.7 fold), fructose 16 bisphosphatase (43.8 fold) although phosphofructokinase expression was increased (2.9 fold). Also down regulated were blood coagulation (coagulation factors II, V, VII, IX, XI, and XII; 22–178 fold). Genes associated with mitochondrial oxidative function that were increased were ATP synthase (2.2 fold), cytochrome C (2.5 fold) and UCP 3 (4.7 fold).

The transcription factor hepatocyte nuclear factor 4α (HNF4α) plays a pivotal role in directing liver gene expression [26]. A significant decrease in HNF4α expression was detected (P<0.01; Figure 1E) by both microarray and qRT-PCR analysis. A number of genes in HNF4α target pathways were significantly down regulated by cold stress including constitutive androstane receptor (CAR; Nr1i3,80 fold)and pregnane×receptor (PXR; NR1i2; 28 fold), both involved in the expression of drug metabolising enzymes [27] and this was accompanied by a significant down regulation of Phase I (CYP2B, CYP2C, CYP3A) and Phase II genes (UDP-glucuronosyl-transferases UGT1A1, UGT1A9). HNF4α is known to coordinate hepatic energy metabolism which might have been expected to alter during cold exposure but there were no significant alterations in target genes in glucose metabolism (e.g PEPCK; GPPase), fat metabolism (e.g. SREB1C; CPT1; FAS, ACC1, SCD1) or mitochondriogenesis (e.g. PGC1α/β; NRF1, TFAM). The only exceptions were significant down regulation (P<0.01) of PPARα (detected by both microarray and qRT-PCR) and the ketogenic enzyme, 3HMGCoA synthetase 1,2, both of which have been reported a HNF4α target genes. The liver plays a major function in lipid transport and HNF4α regulates the expression of a number of lipoproteins that were also suppressed [28].

## Discussion

Cold stimulation increases the plasma concentrations of adrenaline, glucagon, growth hormone, ACTH, insulin and adrenal steroids, TSH [29], [30], as well as activating the sympathetic nervous system. These changes bring about a range of behavioural, physiological and metabolic responses to prevent hypothermia, of which the best described is the increase in adaptive thermogenesis in BAT. Cold induced changes in white adipose tissue and liver are less well understood and here we report alterations in gene expression assessed by microarray to when mice maintained at 22°C were exposed for 24 h at 8°C. It should be noted that recent studies have emphasised that 22°C is still below thermoneutrality so the room temperature acclimated mice are effectively mildly cold stressed [31]. A striking finding in this study is that the GO groups of gene transcripts changing in BAT and liver in response to cold were similar whereas there were far fewer changes in WAT and these did not overlap with either BAT or liver. In BAT and the liver the general trend was for a reduction in the expression of a large number of genes involved in oxidoreductase activity, iron ion binding, endoplasmic reticulum function, lipid metabolic processes and protease inhibitor activity, and far fewer genes were upregulated by the cold challenge. This may represent a mechanism for reducing metabolic reactions so that metabolism could be focussed on the cell thermogenic functions, particularly in BAT, that are essential to prevent hypothermia.

We were able to confirm the cold induced increase in the BAT expression of key genes involved in thermogenesis including UCP1, PGC1α and C/EBPbeta. These changes are known to be coordinated by alteration in sympathetic neurotransmitters which are ligands for membrane G-protein coupled receptors [32]. In agreement with these expectations we were able to observed significant changes in the expression of a number of adrenergic and G-protein related genes in all tissues, in response to cold. Particularly notable in BAT were upregulation of the beta-1 adrenergic receptor and G protein-coupled receptor 120, which has been implicated in the insulin sensitising effect of omega-fatty acids in adipocytes [33].

In liver the transcriptional factor HNF4α expression was significantly decreased 54 fold although by only 1.61 fold in BAT. HNF4α has been shown to regulate a large number of hepatic pathways by interacting with other nuclear proteins such as PPARα and LXR and it was evident that HNF4α and PPARα target genes were particularly well represent in the liver and BAT, down-regulated genes. Expression of nuclear receptor PPARα was significantly decreased three fold in both BAT and liver of cold stressed mice in association with decreased expression of a number of genes regulating cholesterol and fatty acid transport and metabolism which have been proposed to be regulated by PPARα, including Apoa1, Apoa2, Apoc3, Hmgcs2, Cyp7a1. In liver down regulation of this group of PPARα regulated genes was much greater than in BAT with more than 30 fold changes being observed. The nuclear receptor Nr1h4 (farnesoid×receptor) was also down regulated in both BAT and liver during cold exposure. PPARα has been implicated in stimulating fatty acid oxidation so we were surprised to observe down regulation of this gene in both BAT and liver, since both tissue respond to cold stress by increased fat oxidation [34].

The increased expression of DIO II in BAT agrees with previous studies showing that cold increases BAT thermogenesis by converting thryoxine to the active form, triiodothryonine [35]. In the liver we observed down regulation of DIO 1 which is consistent with the cold suppression of hepatic HNF4α which plays a key role in regulating hepatic DIO 1 [36]. However hepatic expression of thyroid binding globulin (Serpin A7) was significantly decreased in liver which has been suggested to be responsible for an increased supply of free thyroid hormones to tissues during cold adaptation [37]. Other thyroid target genes involved in hepatic mitochondriogenesis were not altered.

This study reveals an unexpected importance of down regulation of genes in both BAT and liver, but not WAT, particularly the cytochrome P450 and apolipoprotein gene families, in response to cold exposure. The results of this study support the notion that HNF4α plays a role as a pivotal regulator in the network of nuclear receptors that integrates liver and brown adipose intermediary metabolism in response to acute cold exposure. If the observation of down regulated genes in BAT and liver was converted into changes in protein, it suggests that response to cold stress involves decreased energy expenditure on a range of cellular processes presumably to maximise energy expenditure on heat production.

## Supporting Information

Table S1 **GO term Oxidoreductase activity up regulated and down regulated genes in all tissues.**
(DOCX)

Table S2 **List of genes differentially expressed between the cold and room temperature treatments for brown adipose tissue.**
(XLSX)

Table S3 **List of genes differentially expressed between the cold and room temperature treatments for white adipose tissue.**
(XLSX)

Table S4 **List of genes differentially expressed between the cold and room temperature treatments for liver.**
(XLSX)
