# Supplementary material for: Cold-Induced Changes in Gene Expression in Brown Adipose Tissue, White Adipose Tissue and Liver
Source: PLoS One. 2013 Jul 22;8(7):e68933. doi: 10.1371/journal.pone.0068933 (PMC3718809; doi:10.1371/journal.pone.0068933)
Supplement: Table S1 — GO term Oxidoreductase activity up regulated and down regulated genes in all tissues. (DOCX) [file pone.0068933.s001.docx]

Table S1 GO term Oxidoreductase activity up regulated and down regulated genes in all tissues

| Oxidoreductase activity (Using liver) | |  | Fold change | | Fold change | | Fold change | |
| --- | --- | --- | --- | --- | --- | --- | --- | --- |
| Symbol | Definition_1 | Accession | Brown | Regulation | Liver | Regulation | White | Regulation |
| Maob | monoamine oxidase B | NM_172778.1 | 2.34 | up | 16.19 | up | 2.92 | Up |
| Ndufb4 | NADH dehydrogenase (ubiquinone) | NM_026610.1 | 1.73 | up | 3.75 | up | 1.76 | Up |
| Hsd3b2 | hydroxy-delta-5-steroid dehydrogenase, | NM_153193.2 | 1.32 | up | 6.43 | down | 1.73 | Down |
| Mdh2 | malate dehydrogenase 2, NAD (mitochondrial) | NM_008617.2 | 1.28 | up | 3.34 | up | 1.54 | Up |
| Scp2 | sterol carrier protein 2, liver | NM_011327.1 | 1.25 | up | 9.72 | down | 2.07 | Down |
| Bbox1 | butyrobetaine (gamma), 2-oxoglutarate dioxygenase 1 | NM_130452.1 | 1.22 | up | 58.64 | down | 1.20 | Down |
| Cyp2c39 | cytochrome P450, family 2, subfamily c, polypeptide 39 | NM_010003.1 | 1.22 | up | 12.04 | down | 1.90 | Up |
| Enox1 | ecto-NOX disulfide-thiol exchanger 1 | NM_172813.1 | 1.21 | up | 2.56 | up | 1.00 | Down |
| Cyp2d22 | cytochrome P450, family 2, subfamily d, polypeptide 22 | NM_019823.2 | 1.16 | up | 13.58 | down | 1.02 | Down |
| Qdpr | quinoid dihydropteridine reductase | NM_024236.1 | 1.13 | up | 3.22 | down | 1.04 | Down |
| Rdh20 | retinol dehydrogenase 20 | NM_027301.3 | 1.07 | up | 5.88 | down | 1.07 | Up |
| Akr7a5 | aldo-keto reductase family 7 | NM_025337.2 | 1.05 | up | 3.90 | down | 1.11 | Up |
| Dmgdh | dimethylglycine dehydrogenase precursor | NM_028772.1 | 1.01 | up | 28.75 | down | 1.43 | Up |
| Aldh16a1 | aldehyde dehydrogenase 16 family, member A1 | NM_145954.1 | 1.01 | up | 3.20 | down | 1.16 | Up |
| Uox | urate oxidase | NM_009474.3 | 14.86 | down | 267.13 | down | 1.36 | Down |
| Cyp2d9 | cytochrome P450, family 2, subfamily d, polypeptide 9 | NM_010006.1 | 14.64 | down | 232.58 | down | 1.10 | Down |
| Cyp2c37 | cytochrome P450, family 2. subfamily c, polypeptide 37 | NM_010001.1 | 9.25 | down | 136.25 | down | 1.14 | Up |
| Cyp2c70 | cytochrome P450, family 2, subfamily c, polypeptide 70 | NM_145499.1 | 9.24 | down | 137.75 | down | 1.14 | Down |
| Cyp2d10 | cytochrome P450, family 2, subfamily d, polypeptide 10. | NM_010005.2 | 8.85 | down | 281.15 | down | 1.01 | Down |
| Cyp2a12 | cytochrome P450, family 2, subfamily a, polypeptide 12 | NM_133657.1 | 8.34 | down | 143.86 | down | 1.08 | Down |
| Rdh7 | retinol dehydrogenase 7 . | NM_017473.1 | 7.19 | down | 190.17 | down | 1.36 | Down |
| Cyp8b1 | cytochrome P450, family 8, subfamily b, polypeptide 1 | NM_010012.2 | 5.70 | down | 87.84 | down | 1.51 | Up |
| Hpd | 4-hydroxyphenylpyruvic acid dioxygenase. | NM_008277.1 | 5.68 | down | 164.66 | down | 1.85 | Up |
| Cyp4f14 | cytochrome P450, family 4, subfamily f, polypeptide 14 | NM_022434.1 | 5.55 | down | 153.05 | down | 1.42 | Down |
| Cyp1a2 | cytochrome P450, family 1, subfamily a, polypeptide 2 | NM_009993.2 | 4.99 | down | 214.85 | down | 1.33 | Down |
| Cyp7a1 | cytochrome P450, family 7, subfamily a, polypeptide 1 | NM_007824.2 | 3.35 | down | 45.55 | down | 2.66 | Up |
| 0610012D14Rik | RIKEN cDNA 0610012D14 gene | NM_026690.1 | 3.32 | down | 102.39 | down | 1.08 | Down |
| Cyp2d26 | cytochrome P450, family 2, subfamily d, polypeptide 26 | NM_029562.1 | 2.87 | down | 98.69 | down | 1.10 | Down |
| Fmo5 | flavin containing monooxygenase 5 | NM_010232.3 | 2.12 | down | 23.92 | down | 1.02 | Down |
| Prodh2 | proline dehydrogenase (oxidase) 2 . | NM_019546.4 | 2.08 | down | 133.10 | down | 1.06 | Up |
| Prodh2 | proline dehydrogenase (oxidase) 2 . | NM_019546.4 | 1.67 | down | 81.62 | down | 1.19 | Up |
| Cyp27a1 | cytochrome P450, family 27, subfamily a, polypeptide 1 | NM_024264.3 | 1.66 | down | 6.49 | down | 1.03 | Up |
| Rdh16 | retinol dehydrogenase 16 | NM_009040.1 | 1.62 | down | 15.01 | down | 1.73 | Up |
| Acox2 | acyl-Coenzyme A oxidase 2, branched chain. | NM_053115.1 | 1.56 | down | 60.53 | down | 1.11 | Down |
| Cyp46a1 | cytochrome P450, family 46, subfamily a, polypeptide 1 | NM_010010.1 | 1.35 | down | 22.84 | down | 1.04 | Down |
| Cyp4f15 | cytochrome P450, family 4, subfamily f, polypeptide 15 | NM_134127.1 | 1.25 | down | 33.24 | down | 1.20 | Up |
| Ldhd | lactate dehydrogenase D | NM_027570.3 | 1.22 | down | 2.78 | down | 1.10 | Down |
| Sord | sorbitol dehydrogenase . | NM_146126.1 | 1.05 | down | 5.95 | down | 1.22 | Up |
| Gulo | gulonolactone (L-) oxidase | NM_178747.2 | 1.03 | down | 58.55 | down | 1.08 | Down |
| Gpx8 | glutathione peroxidase 8 (putative). | NM_027127.1 | 1.01 | down | 2.11 | up | 1.74 | Down |
| Pipox | pipecolic acid oxidase . | NM_008952.1 | 1.00 | down | 39.17 | down | 1.03 | Down |
| Pah | phenylalanine hydroxylase. | NM_008777.1 | 1.00 | down | 24.33 | down | 1.24 | Up |
